# Preliminary study on discriminating HER2 2+ amplification status of breast cancers based on texture features semi-automatically derived from pre-, post-contrast, and subtraction images of DCE-MRI

**DOI:** 10.1371/journal.pone.0234800

**Published:** 2020-06-17

**Authors:** Lirong Song, Hecheng Lu, Jiandong Yin

**Affiliations:** 1 Department of Radiology, Shengjing Hospital of China Medical University, Shenyang, Liaoning, P.R. China; 2 College of Medicine and Biological Information Engineering, Northeastern University, Shenyang, Liaoning, P.R. China; INSERM, FRANCE

## Abstract

**Objective:**

To investigate whether texture features extracted from dynamic contrast-enhanced magnetic resonance imaging (DCE-MRI) are associated with human epidermal growth factor receptor type 2 (HER2) 2+ status of breast cancer.

**Materials and methods:**

92 MRI cases including 52 HER2 2+ positive and 40 negative patients confirmed by fluorescence *in situ* hybridization were retrospectively selected. The lesion area was semi-automatically delineated, and a total of 488 texture features were respectively extracted from precontrast, postcontrast, and subtraction images. The Student’s *t*-test or Mann-Whitney *U* test was performed to identify statistically significant features between different HER2 2+ amplification groups. Least absolute shrinkage and selection operator (LASSO) was used to search for the optimal feature subsets. Three machine learning classifiers, logistic regression analysis (LRA), quadratic discriminant analysis (QDA), and support vector machine (SVM), were used with a leave-one-out cross validation method to establish the classification models of HER2 2+ status. Classification performance was evaluated by receiver operating characteristic (ROC) analysis.

**Results:**

Based on the texture analysis with SVM model, the areas under the ROC curve (AUCs) were 0.890 for subtraction images, 0.736 for postcontrast images, and 0.672 for precontrast images, respectively. For LRA model, the AUCs were 0.884, 0.733, and 0.623, respectively. For QDA model, the AUCs were 0.831, 0.726, and 0.568, respectively. LRA and the SVM model with subtraction images reached significantly better performance than the QDA model (*P* = 0.0227 and *P* = 0.0088, respectively).

**Conclusion:**

Texture features of breast cancer extracted from DCE-MRI are associated with HER2 2+ status. Additional studies are necessary to confirm the present preliminary findings.

## Introduction

Breast cancer is a heterogeneous tumor and it is categorized into different molecular subtypes [luminal A, luminal B, human epidermal growth factor receptor type 2 (HER2)-positive, and triple negative (basal like)] [1,2]. The prognosis and survival rates differ significantly among subtypes. HER2 is an important biomarker for determining the molecular subtype of breast cancer, and its expression can usually be determined by immunohistochemistry (IHC). For HER2 scores 0, 1+, and 3+, IHC is accurate for assessing negative or positive status. However, for HER2 2+ patients, further fluorescence *in situ* hybridization (FISH) examination is essential to confirm the gene status. FISH is costly, time-consuming, and requires specialized equipment. HER2-positive cancers contain more HER2 genes and produce more HER2 proteins. Hence, HER2-positive cancers have a tendency to promote rapid growth and division of cancer cells, and stimulate cell proliferation and angiogenesis to provide nutrition [3]. Detection of the amplification status of HER2 2+ by FISH prolongs the time for accurate diagnosis, and the timeliness and accuracy of diagnosis are extremely important for doctors and patients. Therefore, identifying a cost- and time-effective alternative method for distinguishing HER2 2+ positive and negative status would be beneficial [4].

Dynamic contrast-enhanced magnetic resonance imaging (DCE-MRI) is currently the most sensitive modality for the detection of breast cancer [5–8]. MR images contain pixel gray level variations that cannot be evaluated visually, but can be detected using image analysis methods [9]. Tumor quantitative characteristics obtained from MR images are important for differentiating benign from malignant breast lesions [10–12]. Moreover, breast cancers may present specific features in MR images according to molecular subtype [13–19].

Texture analysis (TA) offers a way to calculate mathematical values for texture features, which can be used for characterizing the underlying structures of the observed tissues. The spatial location and signal intensity characteristics of image pixels can evaluated by TA. The application of TA to breast MR imaging studies shows potential value [20–25]. Sun X et al. [2] indicated that texture features from T1 weighted and diffusion weighted images provide a promising approach to predict the molecular subtypes of breast cancer. Chamming’s F et al. [26] reported that kurtosis derived from TA of pretreatment MR images is independently associated with pathological complete response to neoadjuvant chemotherapy in non-triple-negative breast cancer. Ma W et al. [27] showed that DCE-MRI texture features are associated with Ki-67 expression in patients with invasive breast cancer.

The objective of the preliminary study was to evaluate whether texture features are associated with HER2 2+ amplification status in breast cancer. To the best of our knowledge, there are no reports investigating HER2 2+ amplification status using three different machine learning methods respectively based on relatively more kinds of texture features respectively derived from precontrast, postcontrast, and subtraction images of DCE-MRI.

## Materials and methods

This study was approved by the ethics committee of Shengjing Hospital of China Medical University (NO.2019PS175K). The method used in this study was performed in accordance with the approved guidelines. Because of a retrospective study, the requirement for informed consent was waived by the ethics committee of our hospital that approved this study’s protocols.

### Study population

First, two experienced radiologists were employed for the case collection. They were blind to pathological results during reading the breast MRI images independently. They retrospectively assembled an image dataset of 163 patients obtained from February 1, 2018 to December 31, 2018 with our picture archiving and communication system (PACS). All patients underwent breast DCE-MRI examination and had histopathologically confirmed HER2 2+ breast cancer. Patients without FISH examination (n = 38) were excluded from the dataset. Twenty-seven patients who received radiotherapy or chemotherapy before MRI examination were also eliminated from the dataset. Whether there is a motion artifact or not is determined by the consensus of the two radiologists' evaluations. As a result, six patients whose images had substantial motion artifacts or tumors were not definitely visualized were excluded. The final dataset included 92 HER2 2+ breast cancer patients for analysis. The amplification status of HER2 2+ was finally verified using FISH, which is considered as the gold standard in this field.

### DCE-MRI technique

All breast DCE-MRI examinations were performed at our institution using a 3.0 Tesla MR scanner (Signa HDxt, GE Healthcare, USA) with the patient in a prone position and a dedicated eight-channel double-breast coil for signal reception. In each MRI examination, a precontrast series of fat-saturated T1-weighted 3D images based on the VIBRANT-VX technique was initially acquired. After the intravenous injection of a contrast agent (0.5 mmol/ml, Gadodiamide, Omniscan, GE Healthcare, USA; Magnevist, Bayer-Shering Pharmaceuticals) at 4 mL/s with a dose of 0.15 mmol per kg body weight, eight postcontrast scans were acquired. The imaging parameters were as follows: repetition time (TR) = 7.42 ms, echo time (TE) = 4.25 ms, inversion time = 20 ms, flip angle = 15°, slice thickness = 2.20 mm, and spacing between slices = 2.20 mm. The acquisition matrix was 1,024 × 1,024.

### Lesion delineation

**Fig 1:**
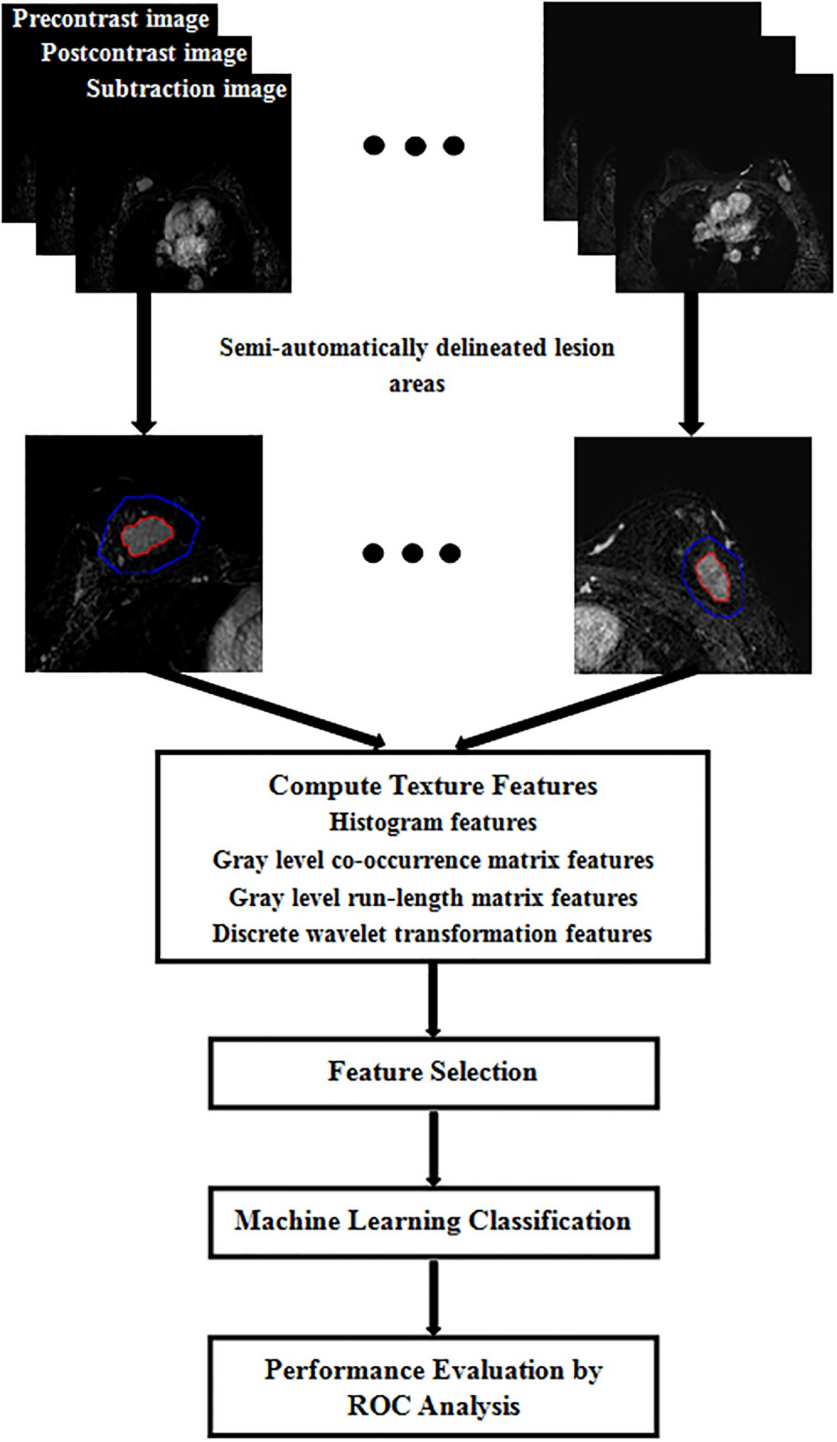
Flowchart of semi-automatic texture analysis adopted in our study.

The slice image with the lesion of maximum diameter was extracted from each volume image for subsequent quantitative analysis. The flowchart of our study is shown in Fig 1. As done by Chang R F et al [22], texture analyses were performed based on axial precontrast T1-weighted images, axial postcontrast T1-weighted images obtained in the fifth phase after contrast material injection, and the subtraction images of these two images. The lesion areas were firstly delineated semi-automatically in the subtraction images using an in-house software programmed with MATLAB 2018a (Mathworks, Natick, MA, USA). The specific segmentation procedures included the following steps [28].

First, a region of interest (ROI) of arbitrary shape was drawn around the lesion area.

Second, the popular segmentation algorithm, Otsu [29], was applied to the ROI pixels. Meanwhile, the segmented image was converted into a binary image with the objective region as 1 and background region as 0.

Third, morphological erosion was applied to the acquired binary image, and the unique but largest eight-connected region was identified.

Finally, morphological dilation was performed in the unique region, and the target region was considered as the lesion area.

Meanwhile, the contour of segmented lesion area on the subtraction slice image was co-registered to the corresponding postcontrast and the precontrast slice images. The results of the semi-automatic segmentation were examined and approved by the two radiologists.

### Texture extraction

TA was only performed for the segmented lesion areas from subtraction, pre- and postcontrast images using the MATLAB 2018a programming platform, respectively. For the texture measurements, the slice images selected from all participants were loaded into the MATLAB in the ".dcm" format. All pixel intensities within the ROI segmented by the semi-automatic method were normalized between μ ± 3σ (μ: mean of image intensity within the ROI; σ: standard deviation), and the range was quantized to 8 bits/pixel. A total of 488 texture features were measured, including histogram-based, gray level co-occurrence matrix (GLCM)-based, gray level run-length matrix (GRLM)-based and discrete wavelet transform (DWT)-based features, as shown in Table 1.

**Table 1:** The features measured with different texture analysis methods.

Methods	Texture features	Number
**Histogram**	Mean, variance, skewness, kurtosis	4
**GLCM**	Autocorrelation (ACOR), contrast (CON), correlation (COR), cluster prominence (CP), cluster shade (CS), dissimilarity (DIS), angular second moment (ASM), entropy (ENT), inverse difference moment (IDM), maximum probability (MP), sum of squares (SOS), sum average (SA), sum variance (SV), sum entropy (SE), difference variance (DV), difference entropy (DE), information measure of correlation (IMC), inverse difference normalized (IDN), inverse difference moment normalized (IDMN)	380
**GRLM**	Run-length non-uniformity (RLN), gray level non-uniformity (GLN), long run emphasis (LRE), short run emphasis (SRE), fraction of image in runs (FIR), low gray level run emphasis (LGRE), high gray level run emphasis (HGRE), short run low gray level emphasis (SRLGE), short run high gray level emphasis (SRHGE), long run low gray level emphasis (LRLGE), long run high gray level emphasis (LRHGE)	44
**DWT**	Harr parameters	20
	Deubechies2 parameters	20
	Symlet4 parameters	20
**Total**		488

GLCM, gray level co-occurrence matrix; GRLM, gray level run-length matrix; DWT, discrete wavelet transformation.

Eighteen GLCM-based texture features were calculated with four distances (1 pixel, 2 pixels, 3 pixels, 4 pixels) in four directions (0°, 45°, 90°, 135°). In the following, (d, 0), (0, d), (d, d), (-d, -d) represent 0°, 45°, 90° and 135°, respectively, and d is the value of distance. For example, S(0,1) CON represented the contrast feature calculated for a distance of 1 and direction of 90°. Eleven GRLM-based texture features were calculated with the distance of one pixel in four directions (0°, 45°, 90° and 135°). DWT-based texture features were calculated for four layers and three directions (horizontal, vertical, diagonal) to produce low and high frequency components with harr, deubechies2 and symlet4 wavelet. For example, harr HD_2 represented the diagonal high frequency component of second layer using harr wavelet.

### Statistical analysis

Statistical analyses were performed with SPSS (version 19.0, Chicago, IL, USA). For categorical variables, a Chi-square test or Fisher’s exact test was performed between HER2 2+ positive and negative groups. A Kolmogorov-Smirnov test for each feature was first performed to confirm whether the samples obeyed a normal distribution [30]. If the distribution was normal (*P* ≥ 0.05), a Student’s *t*-test was used to compare parameters between HER2 2+ positive and negative groups. Otherwise, the Mann-Whitney *U* test was used [30]. *P* < 0.05 was considered statistically significant. The features with statistically significant differences were selected for subsequent analyses. To eliminate the correlations among the significantly different texture features, least absolute shrinkage and selection operator (LASSO) was performed [31].

Three machine learning methods, logistic regression analysis (LRA), quadratic discriminant analysis (QDA), and support vector machine (SVM), were used to establish the classification models for differentiating HER2 2+ status [32]. A leave-one-out cross validation (LOOCV) method, where one sample was used as the test dataset while the remaining samples were utilized as the training dataset, was used for the statistical models to avoid overfitting of the classifiers [33]. That procedure was repeated for each sample.

The receiver operating characteristic (ROC) curve was used to evaluate the classification performance, and the method of DeLong et al. (1988) and binomial exact confidence interval were selected to draw the curves and calculate the areas under the ROC curves (AUC) by the professional statistics software, MedCalc (version 14.10.20, http://www.medcalc.org/). The AUCs were regarded as the indicators of the diagnostic performance. The sensitivity, specificity, and accuracy were provided correspondingly. The *z*-test was applied to measure the statistical significance between the AUCs.

## Results

### Study population

**Fig 2:**
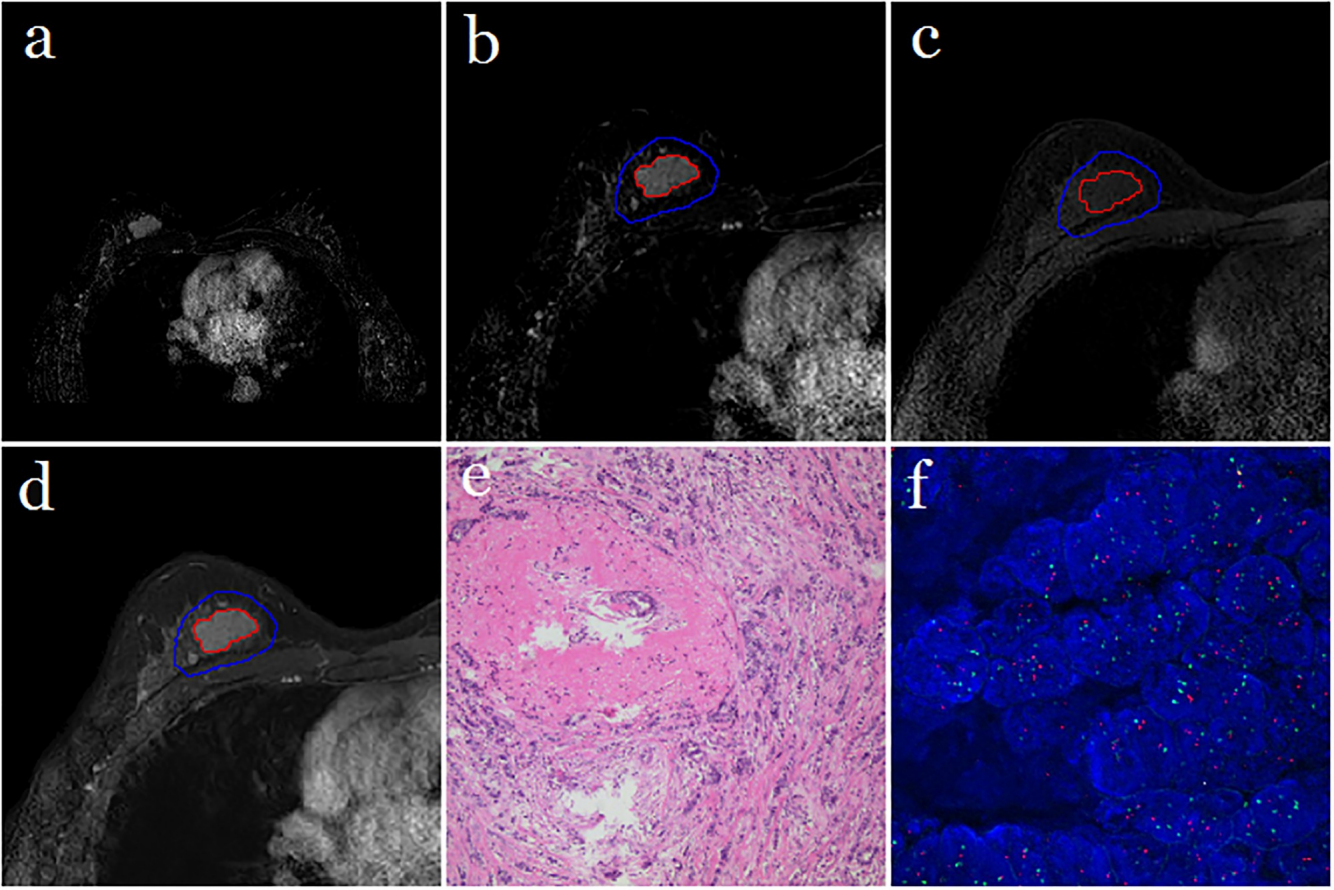
Results obtained from a randomly-selected case with HER2 2+ gene expression. (a) Axial T1-weighted fat-saturated subtraction MR image (regular mass and BI-RADS 4C). (b) Semi-automatic segmentation result of the lesion based on the proposed method by which the color was set to blue for the ROI margin and red for the lesion area margin. (c) Precontrast image covering the same lesion area shown in sub-Figure b. (d) Postcontrast image covering the same lesion area. (e) Histopathological result showing invasive carcinoma of no special type. (f) FISH result showing HER2 negative [HER2/chromosome enumeration probe 17 (CEP17) < 2.0 with the average HER2 signals per cell < 4.0] where red represented HER2 fluorescence signals and green represented CEP17 fluorescence signals.

Ninety-two patients with a median age of 48.5 years (range, 29–69 years) were included in the study group. Patient characteristics are presented in Table 2. Of the 92 HER2 2+ breast cancers, 52 (56.5%) were HER2 2+ positive and 40 (43.5%) were HER2 2+ negative. Fig 2 shows a randomly selected example, where the subtraction image, lesion area on the subtraction image, lesion area on the precontrast image, lesion area on the postcontrast image, histopathological result, and FISH result are presented in sequence.

**Table 2:** Characteristics of 92 patients with HER2 2+ breast cancer.

Characteristics	FISH Results		*P*-Value
	Positive (n = 52; 56.5%)	Negative (n = 40; 43.5%)	
**Age**			0.667^a^
≥ 40 years at diagnosis	41 (58.6%)	30 (41.4%)	
< 40 years at diagnosis	11 (52.4%)	10 (47.6%)	
**Maximum tumor diameter**			0.736^a^
< 20 mm	18 (56.3%)	14 (43.7%)	
≥ 20 mm	34 (56.7%)	26 (43.3%)	
**Gland density**			0.874^b^
Dense type	49 (56.3%)	38 (43.7%)	
Intermediate type	3 (60.0%)	2 (40.0%)	
**Estrogen receptor**			< 0.001^b^
Positive	24 (40.7%)	35 (59.3%)	
Negative	28 (84.8%)	5 (15.2%)	
**Progesterone receptor**			0.001^a^
Positive	26 (44.1%)	33 (55.9%)	
Negative	26 (78.8%)	7 (21.2%)	
**Ki-67**			0.337^a^
≥ 14%	41 (59.4%)	28 (40.6%)	
< 14%	11 (47.8%)	12 (52.2%)	
**Tumor type**			0.515^b^
Ductal carcinoma in situ	1 (50%)	1 (50%)	
Invasive carcinoma of no special type	50 (56.2%)	39 (43.8%)	
Invasive micropapillary carcinoma	1 (100%)	0	

Variable are expressed as frequencies (percentage).

^a^ Variables were tested using the χ^2^test.

^b^ Variables were tested using Fisher’s exact test.

### Texture analysis

**Fig 3:**
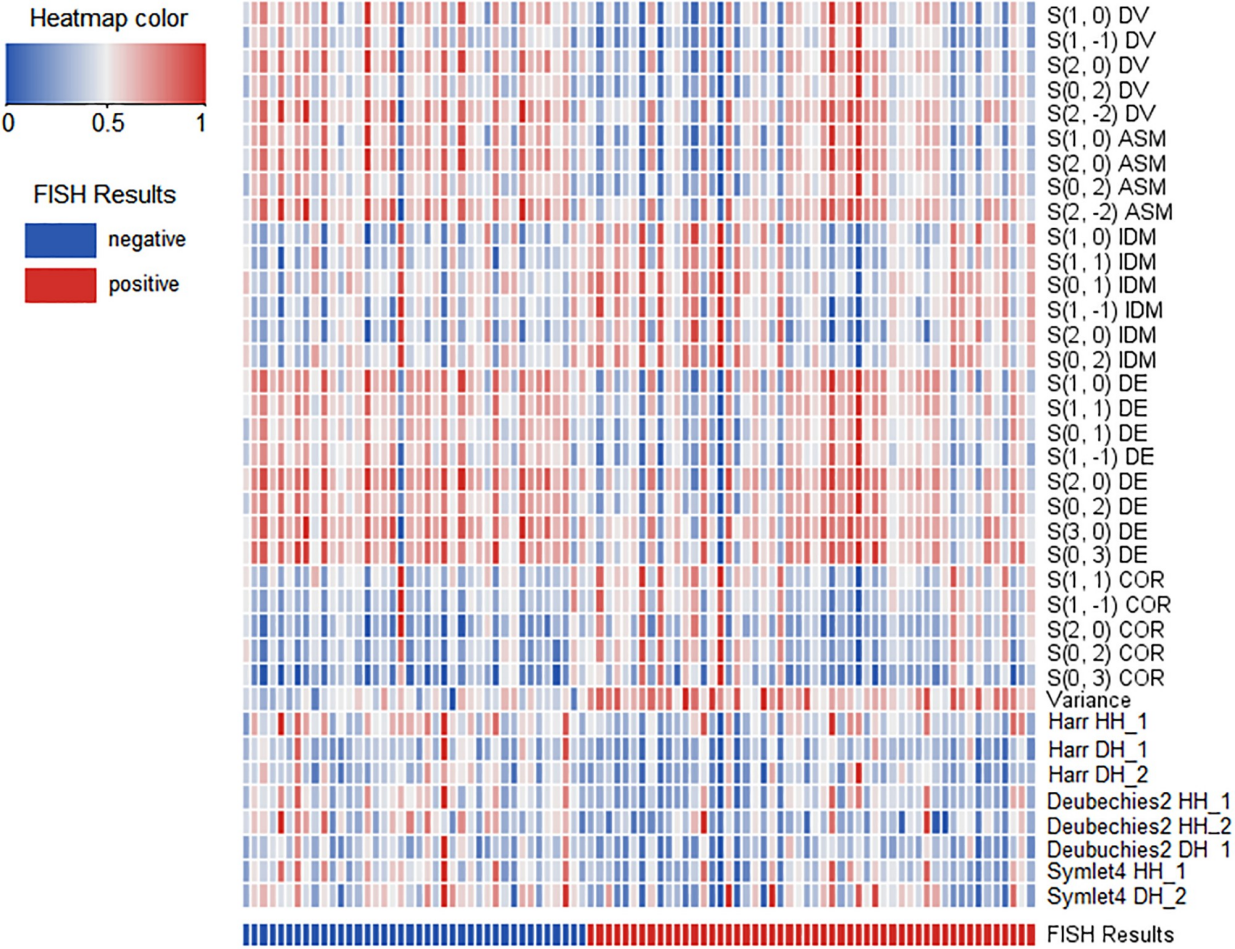
Clustering analysis of the significant features extracted from subtraction images. In the heat map, all 37 significant texture features (presented in different rows) from all 92 patients (presented in each column) were correlated with HER2 2+ status (color coded in the bottom). All features were standardized between zero and one.

**Fig 4:**
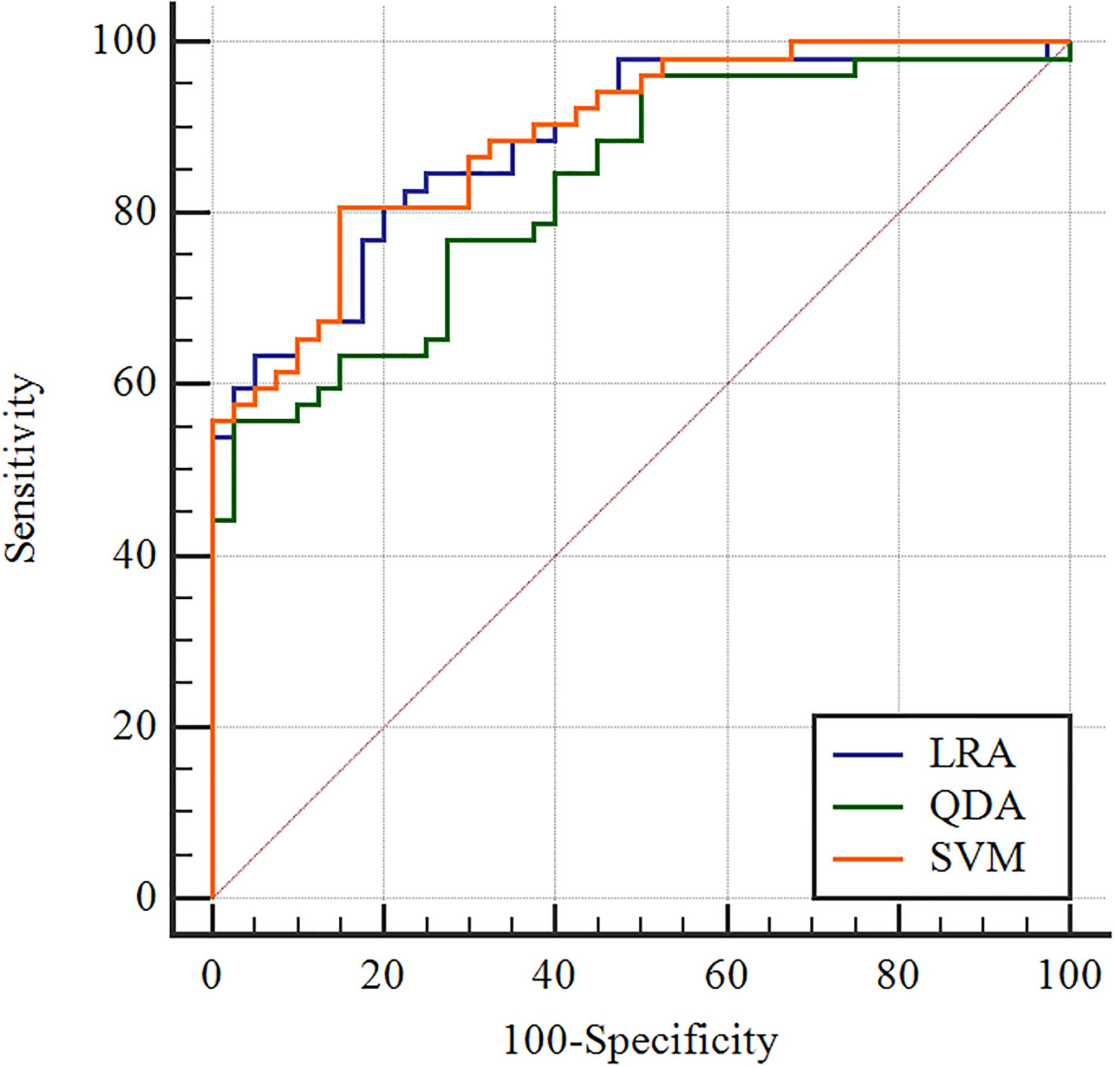
ROC curves of three different classification models based on the subtraction images.

For subtraction images, thirty-seven features presenting statistical difference between HER2 2+ positive and negative patients were selected and illustrated in a heat map (Fig 3). The corresponding details are presented in Table 3. The ROCs reflecting the diagnostic performance of three classification models based on optimal features selected by LASSO are shown in Fig 4. The same analyses were performed for pre- and postcontrast images to discriminate HER2 2+ positive from negative cases. The AUCs from the three types of machine classification based on three kinds of MRI images are listed in Table 4. As shown in the table, three classification models based on the subtraction images achieved the highest performance. The AUCs are 0.884, 0.890 and 0.831, respectively. It can be found that the LRA and the SVM model with subtraction images reached significantly better performance than the QDA model (*P* = 0.0227 and *P* = 0.0088, respectively) (Table 5). There is no significant difference between the AUCs from the LRA and the SVM model. But the SVM model with subtraction images achieved the highest performance with an AUC of 0.890, sensitivity of 80.77%, specificity of 85.00% and accuracy of 82.61%.

**Table 3:** Texture features with statistically significant differences between HER2 2+ positive and negative patients for subtraction images.

Texture features		FISH Results		*P*-Value
		Positive	Negative	
**Histogram**	Variance	22.532 (19.379–25.709)	16.609 (15.511–18.950)	< 0.001^a^
**GLCM**	S(1, 0) DV	8.675±3.621	10.416±3.395	0.021^b^
	S(1, -1) DV	12.676±5.512	14.940±4.638	0.039^b^
	S(2, 0) DV	16.823±6.138	20.192±6.314	0.011^b^
	S(0, 2) DV	16.928±6.204	19.571±5.423	0.035^b^
	S(2, -2) DV	20.575±5.522	23.722±6.478	0.014^b^
	S(1, 0) ASM	4.835±2.738	6.111±2.593	0.026^b^
	S(2, 0) ASM	11.201±4.994	13.961±5.214	0.016^b^
	S(0, 2) ASM	11.291±5.110	13.435±4.472	0.038^b^
	S(2, -2) ASM	14.243±4.553	16.873±5.405	0.013^b^
	S(1, 0) IDM	0.472±0.088	0.424±0.726	0.007^b^
	S(1, 1) IDM	0.390±0.069	0.360±0.060	0.031^b^
	S(0, 1) IDM	0.466±0.070	0.431±0.568	0.013^b^
	S(1, -1) IDM	0.389±0.072	0.352±0.059	0.016^b^
	S(2, 0) IDM	0.331±0.071	0.302±0.057	0.038^b^
	S(0, 2) IDM	0.332±0.060	0.305±0.048	0.025^b^
	S(1, 0) DE	1.642 (1.337–1.759)	1.710 (1.619–1.842)	0.020^a^
	S(1, 1) DE	1.826 (1.584–1.925)	1.890 (1.786–2.013)	0.029^a^
	S(0, 1) DE	1.586±0.207	1.686±0.148	0.011^b^
	S(1, -1) DE	1.795±0.214	1.902±0.165	0.011^b^
	S(2, 0) DE	1.957±0.203	2.072±0.171	0.005^b^
	S(0, 2) DE	1.969±0.185	2.060±0.145	0.013^b^
	S(3, 0) DE	2.087±0.144	2.162±0.147	0.017^b^
	S(0, 3) DE	2.085±0.159	2.158±0.138	0.024^b^
	S(1, 1) COR	0.069±0.026	0.058±0.020	0.029^b^
	S(1, -1) COR	0.068±0.027	0.057±0.023	0.032^b^
	S(2, 0) COR	0.048±0.027	0.035±0.022	0.013^b^
	S(0, 2) COR	0.048±0.023	0.037±0.018	0.013^b^
	S(0, 3) COR	0.030±0.020	0.021±0016	0.026^b^
**DWT**	Harr HH_1	1.619±0.821	2.041±0.923	0.021^b^
	Harr DH_1	0.295±0.124	0.374±0.166	0.011^b^
	Harr DH_2	0.651±0.355	0.811±0.321	0.028^b^
	Deubechies2 HH_1	1.019±0.531	1.312±0.555	0.012^b^
	Deubechies2 HH_4	5.427±5.393	8.184±5.791	0.021^b^
	Deubechies2 DH_1	0.212±0.085	0.258±0.100	0.019^b^
	Symlet4 HH_1	0.814±0.435	1.043±0.474	0.018^b^
	Symlet4 DH_2	0.473 (0.303–0.816)	0.743 (0.512–0.856)	0.024^a^

GLCM, gray level co-occurrence matrix; DWT, discrete wavelet transformation; DV, difference variance; ASM, angular second moment; IDM, inverse difference moment; DE, difference entropy; COR, correlation.

^a^ Variables were tested using Mann-Whitney *U* test, data are median (interquartile range).

^b^ Variables were tested using Student’s *t*-test, data are mean±standard deviation.

**Table 4:** Performance of different classifiers with significant features extracted from MR images.

Classifiers	AUC	SE	95%CI	Sensitivity	Specificity	Accuracy
**Precontrast images**						
LRA	0.623	0.061	(0.516, 0.722)	84.62%	45.00%	67.39%
SVM	0.672	0.058	(0.566, 0.766)	86.54%	45.00%	68.48%
QDA	0.568	0.064	(0.461, 0.671)	80.77%	47.50%	66.30%
**Postcontrast images**						
LRA	0.733	0.052	(0.631, 0.820)	55.77%	82.50%	67.39%
SVM	0.736	0.051	(0.634, 0.823)	84.62%	52.50%	70.65%
QDA	0.726	0.054	(0.623, 0.814)	61.54%	80.00%	69.57%
**Subtraction images**						
LRA	**0.884**	0.034	(0.800, 0.941)	80.77%	80.00%	80.43%
SVM	**0.890**	0.032	(0.808, 0.946)	80.77%	85.00%	82.61%
QDA	**0.831**	0.042	(0.738, 0.901)	55.77%	95.00%	72.83%

AUC, Area of under the ROC curve; SE, Standard error; CI, Confidence interval; LRA, Logistic regression analysis; SVM, Support vector machine; QDA, Quadratic discriminant analysis.

**Table 5:** *P*-values of *z*-test for three classifiers’ AUCs with subtraction images.

Classifiers	LRA	SVM	QDA
**LRA**	/	0.4860	**0.0227**
**SVM**	0.4860	/	**0.0088**
**QDA**	**0.0227**	**0.0088**	/

LRA, Logistic regression analysis; SVM, Support vector machine; QDA, Quadratic discriminant analysis.

## Discussion

HER2 plays a role in the development and progression of several types of human cancer. It has been reported that HER2 positive was associated with favorable pathological features including lower T and N stage and better tumor differentiation in patients with esophageal adenocarcinoma [34]. Jeong J H et al. [35] showed that HER2 amplification is predictive of shorter progression-free survival after cetuximab treatment in patients with metastatic colorectal cancer harboring wild-type RAS and BRAF gene. In fact, the international consensus has been reached that determination of HER2 amplification status is really important not only for treatment options but also for therapeutic efficacy evaluation of breast cancer [36–39].

In most previous studies, lesion areas were manually drawn to be as large as possible around the entire visible tumor by one or two experienced radiologist [40–42]. In this work, a semi-automatic analysis for HER2 2+ status determination was proposed to reduce inter- and intra-operator differences associated with manual methods. The lesion areas were identified by the segmentation algorithm, and texture features reflecting the heterogeneity of the DCE-MRI signal were measured to predict HER2 2+ status in breast cancer. The experimental results demonstrated the diagnostic performance derived from precontrast images was very low for each kind of classifiers. All AUCs (0.623 for LRA, 0.672 for SVM, and 0.568 for QDA) were lower than 0.7. That meant the texture features from precontrast T1WI images presented unsatisfactory effectiveness and were not applicable to determine HER2 2+ status. The diagnostic efficiency from the postcontrast images was moderate, and the AUC values representing diagnostic effectiveness from the three classification methods were also similar (0.733 for LRA, 0.736 for SVM, and 0.726 for QDA). The optimal performances were achieved based on subtraction images, and all AUC values were larger than 0.8. For the three classifiers, SVM is the best, the corresponding AUC was as high as 0.890 which resulted in the highest diagnostic accuracy of 82.61%. In addition, the significant difference was achieved between AUCs respectively derived from QDA and SVM. The above results indicated that the hidden information on the subtracted image was most helpful in identifying the expression status of HER2 2+. That was a very interesting and important finding that was not reported in previous studies. Our findings suggest that texture features may be helpful for determining the molecular subtypes of breast cancer, which is important for predicting prognosis, survival analysis, and decision-making regarding treatment options.

We analyzed the histogram features, GLCM features, GRLM features, and DWT features, which have previously been applied in other oncology fields [43–45]. Features extracted from GLCM characterize the relationship between voxels and their neighborhoods, and reflect the uniformity of the distribution for the image and the heterogeneity of the tumors. The above texture features, such as difference variance, angular second moment, inverse difference moment, difference entropy, and correlation could be applied to differentiate HER2 2+ positive and negative status. These features, which cannot be accurately and reliably evaluated using visual or subjective evaluation methods, can be used as candidate imaging biomarkers for the clinical analysis of breast cancer.

The present study differed from those published previously in several aspects. Most studies investigate texture features using postcontrast T1-weighted MR images, whereas we evaluated both precontrast and subtraction images [46, 47]. Features from subtraction images provided the best results regarding the association with HER2 2+ status. Our study used a semi-automatic segmentation algorithm for lesion area extraction based on subtraction images with MATLAB, whereas most studies draw the ROI manually, which is time-consuming and labor-intensive.

To improve the classification performance, three machine learning methods, LRA, QDA, and SVM, were used in this study independently. The leave-one-out cross validation provided reliable and superior results. Because of the establishment of these models, TA has the potential to be a valuable clinical tool for identifying HER2 2+ status. However, different artificial intelligence methods may achieve better results when the deep learning algorithm is used to investigate the association between texture features and HER2 2+ status in future studies. Additionally, establishing relationships between texture features and the expression of other receptors may be useful.

The present study had several limitations. First, the sample size was relatively small. A large number of extracted texture features and a relatively small sample size may cause classifier overfitting and limit the generalizability of the results. Additional validation studies are necessary. Second, because this study was a single-center study with standardized and uniform MR imaging parameters, the results may not be applicable to other institutions, especially those that use different MR imaging techniques. Moreover, only texture features from tumour ROI were used for predicting HER2 2+ status. In fact, some additional clinical factors, such as ROI/tumour volume, enhanced morphology, time intensity type of contrast agent, quantitative information from diffusion-weighted MR imaging, might also be good predictors in determining the molecular type (estrogen receptor, progesterone receptor, Ki-67, HER2) of breast cancer. Therefore, it is reasonable to believe that the combination of texture features and clinical variables would improve the diagnostic accuracy of HER2 2+. Fourth, TA was only performed with two-dimensional images of the tumors with maximum diameter. Therefore, representation of the entire tumor volume may be limited compared with that in three-dimensional images. Further studies are needed to investigate software programs devoted to TA of three-dimensional volume images. Finally, an important table in radiomics field, radiomics quality score (RQS), was not investigated, which meant that our study might not strictly comply with the requirements of the radiomics workflow [48–50]. As reported by Schick U et al [51], radiomics involves the following execution chain: image acquisition and/or collection, images preprocessing (filtering, registration of sequences, inhomogeneity correction, interpolation, etc.), determination of the volume of interest (manual or semi-automatic), calculation of features (potentially several variants), and finally training and validation of models through statistical analysis (machine learning). In our study, some important steps were lacked, including, external data validation, reproducibility evaluation, and so on, which were also considered to be major challenges for the radiomics field in the previous studies [52].

## Conclusions

In conclusion, the results of this study demonstrated that analysis of texture features extracted from breast MR images is useful for determining HER2 2+ status. More factors such as larger sample size, deep learning algorithms and quantitative MR parameters should be considered to integrate into further studies.
